# MicroRNAs in Vascular Eye Diseases

**DOI:** 10.3390/ijms21020649

**Published:** 2020-01-19

**Authors:** Chi-Hsiu Liu, Shuo Huang, William R. Britton, Jing Chen

**Affiliations:** Department of Ophthalmology, Boston Children’s Hospital, Harvard Medical School, Boston, MA 02115, USA; Chi-Hsiu.Liu@childrens.harvard.edu (C.-H.L.); Shuo.Huang@childrens.harvard.edu (S.H.); William.Britton@childrens.harvard.edu (W.R.B.)

**Keywords:** AMD: biomarker, eye disease, retinopathy, neovascularization, microRNA

## Abstract

Since the discovery of the first microRNA (miRNA) decades ago, studies of miRNA biology have expanded in many biomedical research fields, including eye research. The critical roles of miRNAs in normal development and diseases have made miRNAs useful biomarkers or molecular targets for potential therapeutics. In the eye, ocular neovascularization (NV) is a leading cause of blindness in multiple vascular eye diseases. Current anti-angiogenic therapies, such as anti-vascular endothelial growth factor (VEGF) treatment, have their limitations, indicating the need for investigating new targets. Recent studies established the roles of various miRNAs in the regulation of pathological ocular NV, suggesting miRNAs as both biomarkers and therapeutic targets in vascular eye diseases. This review summarizes the biogenesis of miRNAs, and their functions in the normal development and diseases of the eye, with a focus on clinical and experimental retinopathies in both human and animal models. Discovery of novel targets involving miRNAs in vascular eye diseases will provide insights for developing new treatments to counter ocular NV.

## 1. Introduction

The Human Genome Project from 1990 to 2003 provided, for the first time, complete comprehensive information on human genome sequences with their entire complexity and thereby started the post-genomic era [1,2]. This era is marked in part by extensive studies on non-coding RNAs (ncRNAs), which do not encode for proteins yet still account for more than 98.5% of human genome transcripts [1,2]. While the housekeeping ncRNAs, such as transfer RNAs (tRNAs) and ribosomal RNAs (rRNAs), have already been shown to exhibit relatively clear functions, the regulatory ncRNAs including long non-coding RNAs (lncRNAs), microRNAs (miRNAs), and circular RNAs (circRNAs), once considered “junk” RNAs, have more recently been found to play important roles in a wide variety of physiological and pathological processes, and hence have become attractive targets in deciphering and treating human diseases. Among these regulatory ncRNAs, miRNAs are arguably one of the most widely studied ncRNAs in biomedical research. 

First identified in 1993 [3], miRNAs are a class of naturally occurring small ncRNAs ranging in size from 19 to 25 nucleotides, and their major function is in regulating gene expression at the post-transcriptional level [4]. Since the initial discovery, thousands of miRNAs have been found in various species and the number of miRNAs continues to increase. In the human genome, expression of up to 60% of protein-coding genes may be regulated by miRNAs, indicating their pervasive role in multiple biological processes such as proliferation, apoptosis, differentiation, and development [5,6,7,8]. Importantly, dysregulation of miRNA is found to be involved in many diseases, such as various cancers, cardiovascular diseases, and neurodegenerative disorders. Genetic variation of miRNAs is also linked with several inherited diseases including hearing loss and growth defects. Given their biological importance, miRNAs are currently recognized as novel disease biomarkers and potential therapeutic targets for developing new interventions [8,9,10].

## 2. miRNA Biogenesis and Function

**Figure 1:**
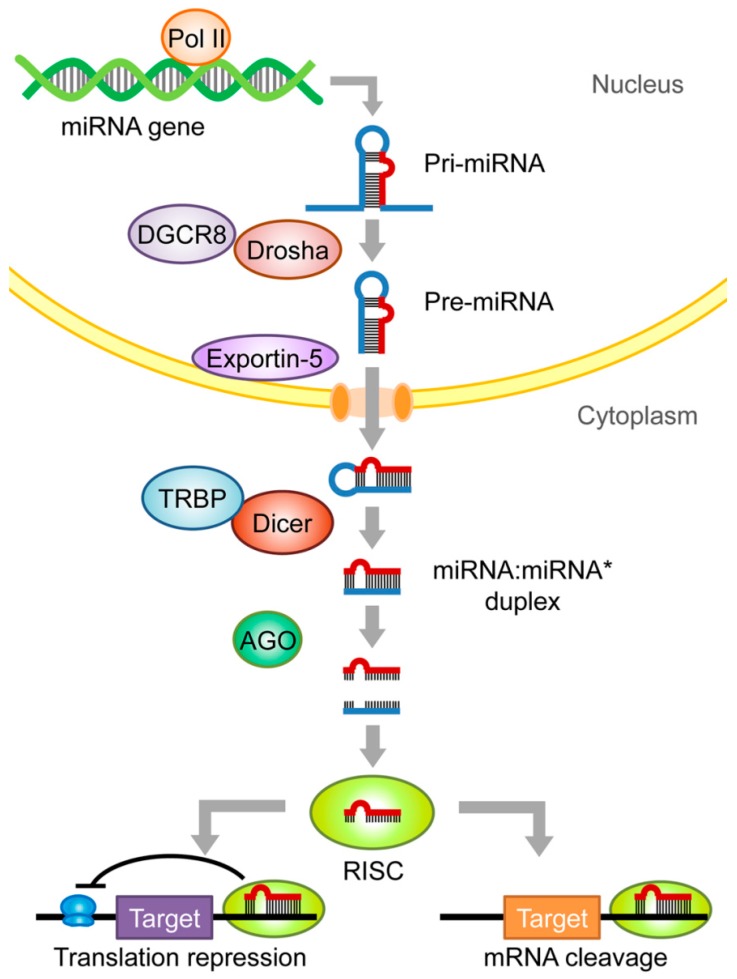
MicroRNA (miRNA) biogenesis. This schematic diagram illustrates the canonical pathway of miRNA biogenesis. The miRNA gene is transcribed by RNA polymerase II (Poly II) to generate the primary miRNA (pri-miRNA) that forms hairpin structures. The long pri-miRNA is then processed by Drosha and DiGeorge syndrome critical region 8 (DGCR8) into the shorter precursor miRNA (pre-miRNA), which is then exported to the cytosol with the help of exportin-5. The pre-miRNA is further cleaved by Dicer and transactivation response element RNA-binding protein (TRBP), yielding the miRNA:miRNA* duplex molecule, which is loaded into argonaute (AGO) to unwind and form the functional RNA-induced silencing complex (RISC). The mature miRNA then binds to the seed sequences on the 3′ untranslated region (3′UTR) of the target mRNA, leading to its translation repression or cleavage and thereby degradation.

The biogenesis of miRNAs starts in the nucleus, where miRNA genes are transcribed primarily by RNA polymerase II (Pol II) into long primary miRNA transcripts (pri-miRNAs), which contain one or more hairpin structures and can be more than 1000 nucleotides long [11]. Pri-miRNAs are then processed by the RNaseIII endonuclease, Drosha, along with its co-factor protein DGCR8 (DiGeorge syndrome critical region 8) into the resulting much shorter precursor miRNAs (pre-miRNAs), usually about 70 nucleotides long, which are then transported to the cytoplasm via exportin-5 [12,13,14,15]. In the cytoplasm, the pre-miRNAs are subsequently cleaved by another RNaseIII endonuclease, Dicer, with assistance from TAR RNA binding protein (TRBP), which together remove the terminal loop, and generate a miRNA duplex [16]. After double-strand RNA unwinding, the mature miRNA strand is selected by the argonaute (AGO) family protein, and assembled into an RNA-induced silencing complex (RISC). This complex may then bind downstream targets to act through translational repression or mRNA cleavage [17,18] (Figure 1). miRNAs function by base-paring to the complementary sequence in the 3′ untranslated region (3′UTR) of target mRNAs to either induce mRNA degradation, promote deadenylation, or reduce translational efficacy [4,19]. Based on these features, miRNAs are considered key mediators of post-transcriptional regulation through fine-tuning gene expression. 

## 3. miRNA as Clinical Biomarkers 

Over the past two decades, numerous studies recognize the clinical value of miRNAs in the diagnosis of virtually all major diseases, including cancers, cardiovascular diseases, and neurodegenerative diseases. The deregulation of miRNAs in cancer was first reported in chronic lymphocytic leukemia in 2002 [20]. Since then, extensive evidence points to largely altered expression levels of miRNAs in various types of cancers; and suggests the immense diagnostic potential of miRNA alteration, specifically circulating miRNAs, as biomarkers [21]. The diagnostic value of many circulating miRNAs has been reported for both chronic and acute leukemia (e.g., *miR-10*, *miR-29*, *miR-31*, *miR-34*, *miR-130b*, *miR-146a*, *miR-148*, *miR-150*, *miR-155*, *miR-181b-5p*, *miR-192*, *miR-203*, *miR-210*, *miR-212*, *miR-328*, *miR-335*, *miR-342*, *miR-375*) [22,23,24,25,26,27], breast cancer (e.g., *miR-9*, *miR-10b*, *miR-21*, *miR-155*, *miR-181a-5p* and *miR-192*) [28,29,30,31,32], lung cancer (e.g., *miR-7*, *miR-25*, *miR-193a-3p*, *miR-214*, and *miR-483-5p*) [33,34,35,36,37], and other human cancers [38], to name just a few. Besides cancer, circulating miRNAs are also implicated in the diagnosis of cardiovascular diseases and neurodegenerative diseases. For instance, *miR-1*, *miR-133a*, *miR-133b*, *miR-145*, *miR-208a*, *miR-208b*, and *miR-499*, may have diagnostic potential for coronary heart disease [39]; whereas four decreased serum miRNAs, namely *miR-141*, *miR-146b-5p*, *miR-193a-3p*, and *miR-214*, are suggested as biomarkers to detect early stages of Parkinson’s disease [40]. In addition, mutations in miRNAs are also associated with inherited diseases, including *miR-96* in progressive hearing loss [41], *miR-184* in familial keratoconus with cataract [42], and the *miR-17~92* cluster in skeletal and growth defects [43]. Together these findings strongly suggest the useful value of miRNAs as clinical biomarkers and diagnostic tools to identify diseases in multiple organs.

## 4. miRNA in the Eye 

**Figure 2:**
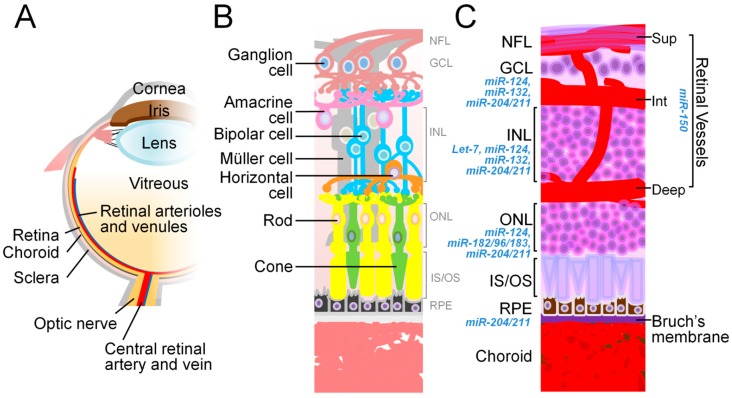
The anatomy of the eye and relevant miRNAs. (**A**) The schematic diagram illustrates the main structures of the human eye. (**B**) The schematic representation of the cell types in the neural retina depicts their cellular connections (including ganglion cells, amacrine cells, bipolar cells, horizontal cells, as well as rod and cone photoreceptors) and supporting cells (Müller cells and RPE). (**C**) A cross section of the eye shows the laminar organization of the nuclear layers (GCL, INL, and ONL), the retinal vasculature, and segments of photoreceptors (IS/OS). The RPE monolayer, with Bruch’s membrane underneath, is located between the neural retina and the choroid complex. miRNAs that regulate the physiological functions or pathological conditions related to each retinal neuronal and vessel layers, and RPE, are listed next to respective histological structure. Deep, deep layer of retinal vessels; GCL, ganglion cell layer; INL, inner nuclear layer; Int, intermediate layer of retinal vessels; IS/OS, inner/outer segments; ONL, outer nuclear layer; RPE, retinal pigment epithelium; Sup, superficial layer of retinal vessels. Figure adapted from “Animal models of ocular angiogenesis: from development to pathologies” by Liu et al. 2017, *FASEB J*, 31(11), p. 4665–4681 [57].

Just like elsewhere in the body, the function of miRNAs has shown increasing relevance in the eye. In the back of the eye, the retina is comprised of a thin layer of neuronal tissue of diverse cell types and is equipped with a highly specialized light-sensing capacity (Figure 2). Fine-tuning gene expression for cell differentiation and function is crucial for vertebrate retinal development and proper vision. Given its major impact on gene regulation, miRNA serves a vital role in the retina throughout development and in eye diseases [44,45,46]. Previous animal studies examining the effects of Dicer-deficiency in retinal development identified the differential expression patterns of many miRNAs in vertebrate neural retinas [47,48,49,50,51,52,53,54,55,56], and developed the ability to categorize retinal cells through specific miRNA signatures (Figure 2).

In the developing eye of several species including mammals, amphibians, and fish, miRNA expression patterns were analyzed using a variety of different approaches [48,52,54,58,59,60,61]. Although the expression profiles of miRNAs vary across species and also differ based on the methods of analysis, some specific miRNAs demonstrate their similar patterns and common roles in retinal development, and in both structural and functional maintenance [62,63,64]. For example, *miR-204*, one of the most abundant miRNAs in the retina, regulates multiple aspects of eye development [60,61]. Ablation of *miR-204* results in abnormal lens formation, altered dorsoventral patterning of the retina, dedifferentiation of the retinal pigment epithelium (RPE), microphthalmia, and coloboma [62,63]. It is strongly expressed in the ganglion cell layer (GCL), the inner nuclear layer (INL), and the RPE [61,65]. In addition, *miR-211*, another member of the *miR-204/211* subfamily, is highly similar to *miR-204* in its sequence, target capability, and expression pattern. Loss of *miR-211* in mice results in progressive cone dystrophy which is accompanied by the degeneration of cone cells and reduced visual responses detected from electroretinogram (ERG) [66]. 

Some miRNAs important for brain neural development also play crucial roles in the retina, as a part of the central nervous system (CNS). *miR-124,* enriched in the vertebrate CNS, is one the most well studied miRNAs in the developing retina [51,52,54,61,64,67,68,69,70,71,72,73,74]. *miR-124* targets *Lhx2*, a transcriptional factor essential for CNS development, and hence it is important in the maturation and survival of both dentate gyrus neurons and retinal cone photoreceptors. *miR-124* is expressed in all retinal neuronal cell layers with the most prominent expression in photoreceptors [46,64]. Abolishing *miR-124a* in mice leads to mislocalization and apoptosis of cone photoreceptors, altered expression of cone-specific genes and reduced photopic ERG [64]. In addition to photoreceptors, *miR-124* is expressed in other retinal neuronal cell layers and contributes to their functions. For instance, in donor eyes from patients with age-related macular degeneration (AMD) and in mouse models of retinal degeneration, *miR-124* exhibits a time-dependent altered expression pattern from the outer nuclear layer (ONL) neurons to the Müller glia in the INL, followed by its eventual depletion at a later stage [75]. *miR-132,* another critical miRNA in brain neural synaptic growth [76], is also important for retinal neurons. As a member of the *miR-132/212* cluster, *miR-132* shapes brain synapse formation and influences visual cortex plasticity [77]. In the eye, *miR-132* promotes axon formation of retinal ganglion cells (RGCs), and is expressed in GCL and INL under the control of brain-derived neurotrophic factor (BDNF) [78]. 

Given the crucial role of the aforementioned miRNAs in ocular and retinal development and their functional conservation across species, it is plausible that altered expression of miRNAs may lead to ocular disorders, including those vascular eye diseases that are characterized by primary abnormalities in retinal blood vessels which control metabolic availability of oxygen and nutrients to impact retinal neurons. 

## 5. miRNA and Angiogenesis 

To understand the roles of miRNAs in vascular eye diseases, here we first summarize the function of miRNAs in angiogenesis. Angiogenesis is the process of new vessels sprouting from existing vessels, which is orchestrated by various angiogenic stimulators and inhibitors, including miRNAs. Angiogenesis plays crucial roles in both physiological development and homeostasis, as the vascular system delivers nutrients to organs and tissues and removes catabolic products [79,80]. Dysregulation of vascular growth is associated with many cardiovascular diseases, cancers, neurodegenerative disorders, and vascular eye diseases [81,82]. Abnormal angiogenesis disrupts the delivery of oxygen and nutrients, which can lead to an unbalanced metabolic status, and result in structural instability and functional loss of affected tissues. Many eye diseases with vascular components are marked by pathologic ocular neovascularization (NV), characterized by a leaky, fragile, and tuft-liked appearance, which may cause retinal hemorrhage and lead to retinal damage and/or tractional retinal detachment, and ultimately result in vision loss in the most severe cases [83]. 

The importance of miRNAs in angiogenesis and endothelial function was first established by analysis of Dicer- and Drosha-deficient mice with defective miRNA biosynthesis [84,85,86,87]. Mouse embryos with a *Dicer* hypomorphic mutation have defective angiogenesis accompanied by reduced miRNA production and dysregulation of angiogenic genes [84,85]. In addition, the knockdown of Dicer and Drosha in human endothelial cells (ECs) suppresses angiogenic functions, including sprouting, tubular formation and migration [86]. Moreover, mice with conditional EC-specific loss of Dicer exhibit reduced vascular endothelial growth factor (VEGF)-driven angiogenesis postnatally, along with an altered set of angiogenic regulating miRNAs [87]. 

Several miRNAs are highly expressed in vascular endothelium, including *miR-126*, *miR-210*, *miR-221/222*, the *miR-17-92* cluster, and the *miR-23-27-24* cluster, as revealed by miRNA profiling in human ECs [88,89,90,91,92,93]. These miRNAs are also called “angiomiRs” for their angiogenesis-related targeting genes [89]. For example, *miR-126* promotes angiogenesis and enhances VEGF signaling in ECs, as it suppresses sprouty-related protein-1 (*Spred1*), a negative factor of Ras-MAP kinase pathway involved in VEGF signaling [90,91,92]. *miR-126* null mouse embryos have vascular abnormalities and a high mortality rate most likely due to SPRED1 induction, and subsequent diminished MAP kinase signaling in response to VEGF [91]. *miR-210*, another angiomiR and hypoxia-induced endothelial miRNA, regulates angiogenesis and cell survival in response to hypoxia [93,94]. Overexpression of *miR-210* increases VEGF-driven EC migration and tube formation by targeting Ephrin-A3, an angiogenic receptor tyrosine kinase [93,95,96]. The *miR-23-27-24* cluster is highly enriched in ECs and vascularized tissues [97,98]. Whereas *miR-23* and *miR-27* act as enhancers of angiogenesis in vascular development and pathological angiogenesis [97,99,100], *miR-24* inhibits cardiac angiogenesis [98]. Similarly, *miR-221/222* is identified as an anti-angiogenic miRNA by targeting *c-Kit*, the tyrosine kinase receptor of pro-angiogenic stem cell factor (SCF). Overexpressing *miR-221/222* in HUVECs suppresses c-Kit and thereby inhibits SCF-mediated angiogenic abilities, such as tubular formation and migration [88]. In addition, the *miR-17-92* cluster is a well-characterized polycistronic miRNA that functions as another intrinsic anti-angiogenic regulator in human ECs [101]. Individual miRNAs of this cluster have the ability to cooperate or work independently to modulate multiple signaling pathways, such as the VEGF, Wnt signaling, and PTEN pathways to impact angiogenesis [101,102,103,104]. Together these findings suggest that miRNAs play critical functions in developmental and pathological angiogenesis and are additionally implicated in vascular eye diseases.

## 6. miRNAs Dysregulation in Neovascular Eye Diseases 

Increasing evidence indicates that miRNAs and their biogenesis machinery may be altered and dysregulated in neovascular eye diseases, such as diabetic retinopathy (DR), age-related macular degeneration (AMD), and retinopathy of prematurity (ROP), suggesting the potential of using miRNAs as biomarkers and targeting them for potential therapeutics. In this section, we discuss some prominent examples of clinical studies on miRNAs dysregulation in vascular eye diseases, focusing on miRNAs as potential biomarkers (Table 1). 

### 6.1. miRNAs in DR

DR is one of the most common microvascular complications of diabetes mellitus (DM), which is now recognized as a global epidemic. In the Western world, DR is a leading cause of vision impairment, particularly among individuals of working-age, and poses a significant economic and life quality burden on patients and society [132,133,134]. To find ways to alleviate this burden, it is important to first understand the biochemical basis underlying DR pathogenesis. Hyperglycemia induces alteration in cellular metabolism and causes oxidative injury. Prolonged exposure to hyperglycemia and metabolic changes leads to microvasculature damage in the retinas of diabetic patients. Progressive retinal ischemia eventually stimulates the expression of hypoxia-induced growth factors, such as VEGF, that promote retinal NV [135]. Retinal NV can then cause vitreous hemorrhage or tractional retinal detachment to result in severe vision loss [135,136]. Other major events involved in DR pathogenesis are the breakdown of the blood-retinal barrier and the consequent vascular leakage and thickening of the retina [137]. DR develops in approximately 50% patients with Type 1 diabetes mellitus (T1DM) and over 40% of patients with Type 2 diabetes mellitus (T2DM) by the first decade of incidence [138]. 

In addition to diagnosing DR through histological examination of the fundus vasculature, circulating miRNAs were found to play critical roles in DR development, suggesting that miRNAs could also serve as new biomarkers in detecting or predicting the progress of retinopathy and furthermore, the overall progress of DM [139,140,141]. Analysis of circulating miRNAs from serum or plasma samples of DM patients both with and without DR showed altered expression levels of many miRNAs throughout different patient populations (e.g., ages, type of DM, years after onset, etc.,) [142]. Among them, *miR-27b* and *miR-320a* are the two miRNAs mostly associated with the risks of DR in T1DM [117]. *miR-221*, an anti-angiogenic miRNA, is significantly altered in DM and is involved in the DM physiopathology and macrovascular complications associated with T2DM [113,114,115,116]. In addition, other circulating miRNAs including *miR-12*6 [105,106,107], *miR-150* [109], *miR-155* [110], and *miR-200b* [111] are also dysregulated in DR patients, as well as in pre-clinical animal models of DR [143,144,145,146,147,148,149,150,151,152]. These findings indicate the complex regulation of miRNAs in DR and the potential of miRNAs as biomarkers and/or therapeutic targets for treating DR.

### 6.2. miRNAs in Neovascular Age-Related Macular Degeneration (wet AMD)

AMD is a leading cause of irreversible loss of central vision in the elderly. Approximately 10–18% of individuals between 65 and 75 will lose some central vision as a result of AMD, while this number increases to 30% for those aged 75 or older [153]. There are two major clinical types of AMD: Atrophic (dry form) AMD with photoreceptor and RPE atrophy; and neovascular (wet from) AMD which is characterized by pathologic subretinal vessels originating from the choroid, i.e., choroidal neovascularization (CNV), the hallmark of wet AMD. Although only 10–20% of AMD patients develop wet AMD, this form of the disease accounts for approximately 80% of severe visual loss in AMD cases [154]. Central vision loss occurs when pathological choroidal neovessels protrude into the subretinal space and subsequently leak blood and cause exudates and hemorrhagic detachment of the retina, thereby resulting in irreversible photoreceptor damage [155]. 

As a complex, multifactorial, and progressive disease, AMD is linked with both genetic (including complement) and environmental risk factors [155]. Certain miRNAs associated with the complement factor H (CFH)-mediated inflammatory degeneration and neovascularization are dysregulated in the circulating blood or ocular tissues isolated from AMD patients [118,121,156,157]. Two studies identified differential sets of miRNAs altered in plasma collected from wet AMD patients compared to healthy subjects [118,156]. In one study, 16 miRNAs were found to be dysregulated out of 384 miRNAs screened in wet AMD patient plasma samples using quantitative real-time PCR-based methods, with 10 of the 384 miRNAs being exclusively expressed in the wet AMD patient group [118]. Another similar study using next-generation sequencing identified that 3 out of 203 circulating miRNAs were significantly altered in plasma from wet AMD patients vs. non-AMD controls [156]. Moreover, miRNA microarray screening found 23 out of 337 miRNAs were upregulated in the serum from both dry and wet AMD patients vs. non-AMD cohorts. Among them, only 3 miRNAs were expressed at significantly higher levels in the serum of patients with wet AMD [119]. The difference in miRNA profiles from these studies may reflect the variation in their miRNA screening methodology, the nature of samples (plasma vs. serum), the diverse patient population and the different inclusion criteria. 

Some miRNAs are altered in both AMD patients and pre-clinical models of AMD, including *Let-7*, *miR-126*, and *miR-21*, all of which are implicated in angiogenic pathways [118,119,120,121]. The *Let-7* family, upregulated in AMD patients [118,119], is pro-angiogenic and acts through the inhibition of anti-angiogenic factors tissue inhibitor of metalloproteinase-1 (TIMP-1) and thrombospondin-1 (TSP-1) [84,86,120]. *miR-126* and *miR-21*, both angiomiRs [89,90,91,92,126], are downregulated in the blood samples of AMD patients [118,121], suggesting the dysregulation of angiogenic effects in these patients. Additionally, in experimental models of CNV, *miR-126* regulates CNV lesion size [122,123]. These studies revealed the emerging role of miRNAs in AMD and the possibility of targeting miRNAs for suppressing CNV in neovascular AMD.

### 6.3. miRNAs in Retinopathy of Prematurity (ROP)

ROP is an ocular disease associated with abnormal retinal vascular development that occurs in premature infants and contributes to 6–18% of blindness in children in the developed countries [158,159,160]. ROP is a two-phase disease, beginning with incomplete retinal vessel growth after premature birth, which results in a peripheral avascular zone. As the infant matures, increasing metabolic activities of the peripheral avascular retina cause tissue ischemia and hypoxia. This stimulates a second phase of hypoxia-driven pathological vessel proliferation. In severe cases, pathologic neovessels in the second phase can cause tractional retinal detachment, ultimately leading to blindness [161,162,163]. Current ablation treatments may substantially reduce the incidence of blindness by 25% and improve long-term outcomes in infants with severe ROP. However, these treatments do not address the underlying causes of ROP or other comorbidities, including the failure of normal neuronal and vascular development [163,164,165]. Whether miRNA dysregulation contributes to ROP development has been a subject of recent studies assessing the diagnostic and therapeutic potential miRNAs as novel ROP biomarkers.

Plasma miRNAs were evaluated in premature infants with ROP and compared to preterm infants without ROP in a recent study using high-throughput quantitative real-time PCR [127]. Four out of 46 plasma miRNAs were significantly altered in ROP patients, with *miR-23a* and *miR-200b-3p* being upregulated and *miR-27b-3p* and *miR-214-3p* being downregulated [127]. *miR-23a* represses anti-angiogenic genes, such as sprouty2 (*Spry2*) and semaphorin6A (*Sema6A*), and *Sema6D* [97], and hence might be pro-angiogenic in ROP pathogenesis. Expression of *miR-200b* correlates with VEGF expression [128], and is an angiogenic regulator targeting *Ets-1* in ECs [112]. On the other hand, *miR-27b* and *miR-214* are anti-angiogenic factors as they inhibit VEGF family protein expression [129,130,131]. Dysregulation of these miRNAs in ROP is consistent with their potential roles in mediating pathological angiogenesis in ROP development. 

Profiles of miRNAs are evaluated in several pre-clinical animal models of ROP. The miRNA expression patterns in different models vary widely, and may depend on a number of factors including animal species (mice vs. rats), the oxygen condition, time point of tissue collection, and analysis methods. Some miRNAs exhibit dramatically varied expression patterns in different models, including *miR-126* [144,166], *miR-145* [167,168], *miR-150* [72,167,169], and *miR-155* [170]. The function of these miRNAs as ocular angiogenic regulators are discussed in detail in the next section.

## 7. Dysregulated miRNAs in Experimental Models of Pathological Ocular Angiogenesis

Expression patterns of miRNAs were investigated in several animal models of ocular NV that mimic pathological features of human vascular eye diseases. Of particular relevance to this review is the oxygen-induced retinopathy (OIR) model, mimicking pathological retinal NV in ROP and DR, and the laser-induced CNV model, mimicking wet AMD. By exposing the newborn experimental animals (rodents, in most cases) to continuous hyperoxic or cycling oxygen conditions, the OIR model reliably reproduces the phenotypes of ROP—characterized by an initial phase of vaso-obliteration and a subsequent phase of hypoxia-induced NV [171,172]. As the current diabetic models fail to consistently develop proliferative retinopathy in rodents, the OIR model also serves as a platform to facilitate the investigation of the ischemic angiogenesis aspect of DR [57]. For wet AMD, the rodent model of laser-induced CNV is the most standard and widely used animal model for investigating many aspects of choroidal angiogenesis. In this model an argon laser is used to induce rupture of the Bruch’s membrane, which increases pro-angiogenic and inflammatory factors and stimulates new choroidal vessels growth into the laser-injured subretinal areas to form CNV [173,174,175]. The pioneering work in the laser-induced CNV and the OIR models has laid the experimental foundation for establishing the therapeutic value of anti-VEGF therapies as these are useful models for investigating the mechanisms of NV and evaluating novel anti-neovascular therapies [176,177], including the role of miRNAs. Some examples of well-characterized miRNAs that regulate pathological ocular angiogenesis in experimental models are reviewed here. 

### 7.1. miR-126

*miR-126* is one of the angiomiRs implicated in the regulation of angiogenic factors including VEGF and FGF for vascular growth, and regulates embryonic angiogenesis and cardiac angiogenesis [91,92]. *miR-126* exhibits significant downregulation in the choroids of mice with laser-induced CNV [123], as well as in rodent OIR retinas and choroids [144,166]. Moreover, *miR-126* knockout mice have vascular lesions in the peripheral areas of choroids in mature adults, delayed choroidal vascular development, and focal choroidal vascular atrophy in aged mice [178]. In OIR mice, *miR-126* supplementation inhibits retinal neovascularization and blood-retinal barrier breakdown [144,179]. Overall these findings indicate that *miR-126* is required for maintaining ocular vasculature integrity in pathological conditions.

### 7.2. miR-132

In the mouse models, *miR-132* plays a crucial role in promoting angiogenesis by targeting *Rasa1* (encoding p120RasGAP) [180,181,182,183]. Inhibition of *miR-132* in the mouse models of OIR and retinal angiomatous proliferation (RAP) promotes EC quiescence and prevents NV by enhancing the expression of p120RasGAP [181]. Inhibition of *miR-132* reduces EC function and suppresses growth factor-mediated developmental retinal and tumor angiogenesis in vivo and in vitro [182]. Furthermore, silencing *miR-132* also suppresses corneal angiogenesis after eye infection with herpes simplex virus [184]. Beyond vascular endothelium, *miR-132* is also expressed in the eye by RGCs and is up-regulated in response to BDNF [78]. These findings suggest the angiogenic functions of *miR-132* in neovascular ocular diseases and its additional function in retinal neuronal health. 

### 7.3. miR-145

**Figure 3:**
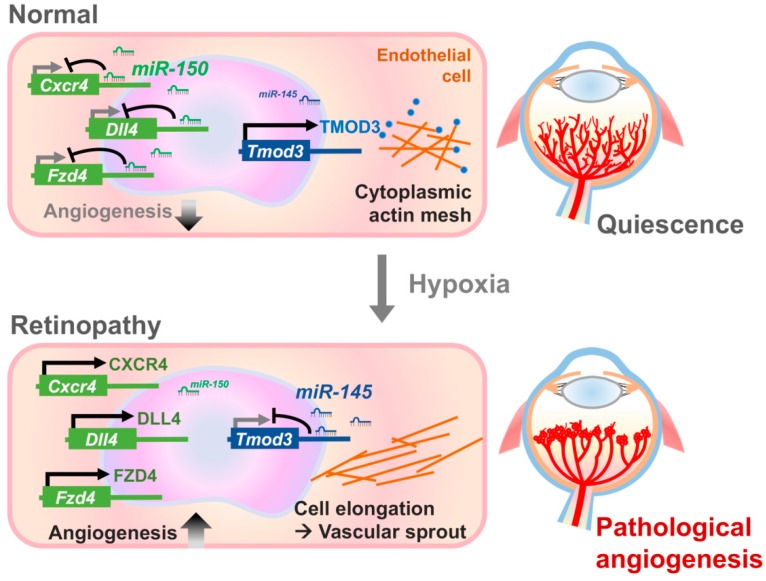
Targeting *miR-150* and *miR-145* in experimental retinopathy. This illustration uses *miR-150* and *miR-145* as the two examples to depict the effects of miRNAs on normal and pathological ocular angiogenesis. In normal retinal vessels, the endothelial-enriched *miR-150* suppresses expression of its downstream angiogenic genes, such as *Cxcr4*, *Dll4,* and *Fzd4*, resulting in reduced angiogenic effects. On the other hand, normal endothelial cells have low levels of *miR-145*, which induces target gene TMOD3, allowing its binding to the pointed end of acting filaments, stabilizing the cytoplasmic actin mesh. High levels of *miR-150* and low levels of *miR-145* in normal retinal vessels both function to maintain quiescence of retinal vessels. On the other hand, in retinopathy, decreased expression levels of *miR-150* in pathological neovessels results in upregulation of its angiogenic targets—CXCR4, DLL4, and FZD4, leading to increased angiogenesis and formation of pathologic neovascularization. Retinal hypoxia in the retinopathy condition also causes the upregulation of *miR-145*, leading to repression of *Tmod3*, releasing the capping of actin filaments. This alteration in actin dynamics and architecture leads to increased endothelial cell angiogenic function, and thereby enhanced pathological angiogenesis. Figure adapted from “*Endothelial microRNA-150 is an intrinsic suppressor of pathologic ocular neovascularization*” by Liu et al. 2015, *PNAS,* 112(39), p. 12163–12168 [169]; and “*MicroRNA-145 Regulates Pathological Retinal Angiogenesis by Suppression of TMOD3*” by Liu et al. 2019, *Mol Ther Nucleic Acids,* 16, p. 335–347 [168].

*miR-145* is co-transcribed with *miR-143* as a cluster, which is generally considered as a tumor suppressor cluster in cancer cells [185]. However, in the mouse model of lung adenocarcinoma, tumor-specific deletion of *miR-143/145* resulted in diminished angiogenesis; whereas overexpression of *miR-143/145* stimulated EC proliferation in the tumor mass [186], indicating a surprising pro-tumor and pro-angiogenic function of *miR-143/145,* likely reflecting its diverse role in a context-dependent manner. Mice with systemic knockout of *miR-143/145* are viable and show no overt abnormalities in cardiac structure and vascular smooth muscle cell differentiation [187]. In the OIR model, *miR-145* is significantly upregulated in the retinas at P17 when compared to the age-matched normoxic control mice [167]. Intravitreal injection of *miR-145* inhibitors suppresses NV in OIR. Moreover, modulation of *miR-145* in vitro alters human retinal microvascular endothelial cell (HRMEC) angiogenic functions by targeting tropomodulin 3 (*Tmod3*), an actin-capping protein. *miR-145* may thereby influence angiogenesis in ocular neovascular diseases through the modulation of the cytoskeletal architecture dynamics, and EC morphological changes [168] (Figure 3). Other studies also showed that *miR-145* in retinal ECs may attenuate oxidative stress and inflammation induced by high-glucose, further supporting its role in DR [188]. Within the eye, *miR-143/145* cluster also regulates intraocular pressure through the regulation of actin dynamics and trabecular meshwork contractility [189]. *miR-145* also promotes ganglion cell survival in DR [190]. These findings demonstrate multiple roles of *miR-143/145* in various ocular cell types and eye diseases.

### 7.4. miR-146a

*mR-146a* has been linked to the innate immune response, inflammation, and age-related neurodegenerative disorders [191]. Samples from patients with wet AMD and mouse retinas with selective glial cell ablation showed an upregulation of *miR-146a* and an involvement in CFH-mediated inflammation [124,125]. In the pre-clinical models of DR, *miR-146a* is upregulated in the retinal ECs with transactivation by nuclear factor-kappaB (NF-κB). Upregulation of *miR-146a* exerts negative regulation in multiple pathways of NF-κB activation, which suggests its correlation to inflammatory responses in DR [148,192,193,194,195,196,197]. Specifically, *miR-146a* upregulates inflammatory cytokines in the diabetic retina and kidney [197], protects HRMECs [194], reduces retinal microvascular leakage, and improves visual function in diabetic rats [198]. Moreover, diabetes induces rhythmic dysregulation of *miR-146a* and its inflammatory genes in human retinal endothelial cells [195]. These findings all point to the potential implication of *miR-146a* in DR development. 

### 7.5. miR-150

*miR-150* is a well-studied miRNA which was initially identified by its regulatory effects in lymphocyte development and differentiation [199,200,201]. Monocytic-secreted *miR-150* influences angiogenesis in cancer and diabetes by modulating target gene expression in recipient ECs [202,203]. In the retina, *miR-150* is enriched in retinal ECs more than in any other nuclear layers [169]. In OIR, *miR-150* is substantially reduced in OIR mouse retinas with specific downregulation in OIR neovessels, and regulates expression of several angiogenic factors, such as *Cxcr4* (C-X-C chemokine receptor type 4), *Dll4* (Delta like ligand 4) and *Fzd4* (Frizzled-4) [72,169] (Figure 3). Treatment of *miR-150* in vivo, via intraocular injection into the OIR mice, or in vitro, by transfection into HRMECs, demonstrated that *miR-150* reduces pathological NV and regulates EC angiogenic functions in a VEGF-independent manner by targeting *CXCR4*, *DLL4*, and *FZD4* [169]. Furthermore, *miR-150* knockout mice show increased size of laser-induced CNV lesion [169], suggesting the role of *miR-150* as an intrinsic inhibitor of pathological ocular angiogenesis. Similarly, *miR-150* deletion leads to increased pulmonary angiogenesis in a hyperoxia-induced lung injury model [204], and exacerbates high fat diet-induced retinal NV in diabetic mice [149], suggesting an overall protective role of *miR-150* against pathological angiogenesis.

### 7.6. miR-155

*miR-155* is significantly upregulated in retinas of several ocular disease models, including the mouse OIR model [167,170], the rat models of light-induced retinal degeneration [205] and streptozotocin (STZ) -induced diabetes [148], as well as in human patients with AMD [124]. *miR-155* is a HIF-dependent miRNA and its deficiency results in the reduction of the avascular area and NV in the mouse OIR model [150,170]. By targeting *CCN1*—a cysteine-rich and integrin-binding matricellular protein, upregulated *miR-155* disturbs the normal retinal vessel growth in mice [170]. In addition to regulation of angiogenesis, *miR-155* is also involved in inflammatory and immunomodulatory signaling pathways, which are of crucial importance in pathological angiogenesis [206]. In the preclinical model of STZ-induced DR, *miR-155* was identified as a NF-κB- and VEGF-responsive miRNA [148]. *miR-155* has also been shown to regulate *CFH* in AMD [124], further supporting its role in ocular angiogenesis and inflammation in eye diseases.

### 7.7. miR-21

*miR-21* plays an important role in the regulation of angiogenesis, tumor growth and metastasis, as well as in cardiac hypertrophy [126,148,207,208]. This miRNA is downregulated in the plasma of wet AMD patients [118]. As such, *miR-21* may play an important role in AMD pathogenesis for its involvement in the regulation of vascular growth, as exhibited by its high expression in retinal ECs [126]. This notion is supported by the fact that overexpression of *miR-21* reduces CNV lesions in the laser-induced CNV mice. In addition, stimulated expression of *miR-21* inhibits cultured EC proliferation and migration by targeted inhibition of *RhoB*, which controls the dynamics of actin-filament and thereby affects the EC function [126]. However, in the rat diabetic model, *miR-21*, as well as *miR-146* and *miR-155*, are upregulated in the retinas and retinal ECs along with NF-κB and/or VEGF activation [148]. In the retinas of leptin receptor-deficient (*db/db*) mice, *miR-21* was also significantly upregulated while its target gene *PPARα* (peroxisome proliferator-activated receptor-α), was downregulated [209]. These different findings from several animal models may reflect an underlying difference among various eye disease models or assay conditions, yet together indicate a potential disease-modifying effect of *miR-21*. 

## 8. Conclusions

In summary, miRNAs are potent effectors in the post-transcriptional regulation of gene activity and play an important role in the modulation of retinal homeostasis and diseases including vascular eye diseases. Although the miRNA expression profiles from different experimental models of ocular angiogenesis differ in a disease- and model-dependent manner, these studies provide valuable clues to understanding the functions of dysregulated miRNAs in retinopathies. Furthermore, dysregulation of specific miRNAs can be utilized to identify potential miRNA candidates for therapeutic intervention. With expanding knowledge of miRNA profiles and their molecular mechanisms in eye development and ocular diseases, miRNAs can be harnessed for their capacities as biomarkers and their potential to be targeted for treating neovascular ocular diseases. In fact, the emerging miRNA therapeutics with its ability to target multiple pathological target genes may likely yield one of the most exciting breakthroughs in the current treatment options for ocular diseases. With the current surge in omics research providing vast amounts of datasets, identification of critical miRNA targets for drug development presents considerable potential for generating such novel therapies for vascular eye diseases. 

## Figures and Tables

**Table 1:** Selected miRNAs associated with DR, wet AMD, and ROP.

Diseases	miRNAs	Effects	miRNA Targets	Reference
**DR**	*miR-12*6	Downregulated in serum of T1DM and T2DM patients	*SPRED-1, PIK3R2, VECAM-1*	[91,92,105,106,107,108]
	*miR-150*	Downregulated in plasma of T1DM with DR	n/a	[109]
	*miR-155*	Upregulated in blood samples of T2DM patients with DR	*TGFB*	[110]
	*miR-200b*	Downregulated in serum of patient with DR	*ETS-1, VEGF-A*	[111,112]
	*miR-221*	Upregulated in serum of T2DM patients	n/a	[113,114,115,116]
	*miR-27b*	Associated with incidence and progression of T1DM by analyzing serum miRNA	*SEMA6A, THBS-1*	[117]
	*miR-320a*	Associated with incidence and progression of T1DM by analyzing serum miRNA	*NRP1*	[117]
**Wet AMD**	*Let-7*	Upregulated in blood samples of AMD patients	*TIMP-1, TSP-1*	[84,86,118,119,120]
	*miR-126*	Downregulated in blood samples of AMD patients	*KDR, SPRED-1, VEGF-A*	[89,90,91,92,118,121,122,123]
	*miR-146*	Upregulated in retinal tissues of AMD patients	*IRAK1, TNFA*	[124,125]
	*miR-21*	Downregulated in blood samples of AMD patients	*RHOB*	[89,118,121,126]
**ROP**	*miR-23a*	Upregulated in plasma of ROP patients	*ISM1, SEMA6A, SEMA6D, SPRY2*	[97,127]
	*miR-200b*	Upregulated in plasma of ROP patients	*ETS-1, VEGF-A*	[112,127,128].
	*miR-27b*	Downregulated in plasma of ROP patients	*VEGF-B, VEGF-C*	[72,127,129,130]
	*miR-214*	Downregulated in plasma of ROP patients	*ANG, HIF1A, QKI*	[72,127,131]

AMD, age-related macular degeneration; DR, diabetics retinopathy; n/a, not applicable; ROP, retinopathy of prematurity; T1DM, Type 1 diabetes mellitus; T2DM, Type 2 diabetes mellitus.
